# 2546. Administration of Crushed Posaconazole Delayed Release Tablets Failed to Achieve Therapeutic Levels

**DOI:** 10.1093/ofid/ofad500.2163

**Published:** 2023-11-27

**Authors:** Nicholas Kane, Rachel Rikard, Katie McCrory, Ashley H Marx

**Affiliations:** UNC Medical Center, Chapel Hill, North Carolina; UNC Medical Center, Chapel Hill, North Carolina; UNC Medical Center, Chapel Hill, North Carolina; University of North Carolina Medical Center, Chapel Hill, North Carolina

## Abstract

**Background:**

Posaconazole (POS) delayed release tablets (DRTs) are utilized for prophylaxis or treatment of invasive fungal infections. Other POS formulations are limited by impaired absorption, lack of indication for adult patients, and need for intravenous (IV) access. Small retrospective case series have demonstrated success with administration of crushed POS DRTs via enteral feeding tubes (EFTs). In October 2022, UNC Health permitted administration of POS DRTs via EFTs. This study aims to evaluate POS levels in patients that received crushed POS DRTs via EFTs.

**Methods:**

This single-center, retrospective cohort study included patients that received crushed POS DRTs via EFTs with concurrent POS therapeutic drug monitoring (TDM). The primary outcome was achievement of serum POS level per indication of prophylaxis ( > 700 ng/mL) or treatment ( > 1000 ng/mL). The secondary outcome was the proportion of patients whose therapy was altered in response to POS level.

**Results:**

A total of 10 patients were included (Table 1), with both prophylaxis (40%) and treatment (60%) indications. Most were initiated on oral or IV POS (80%). Crushed POS DRTs were administered via nasogastric (70%), post-pyloric (20%), and nasoduodenal (10%) routes. Therapeutic levels with crushed POS DRTs occurred in three patients (30%) and only two patients (20%) maintained therapeutic levels (Figure 1). Of the patients that transitioned from IV or oral POS to crushed POS DRT that maintained the same POS dose, 75% experienced a decrease in their serum POS level. Based on the initial crushed POS DRT level, three patients transitioned to IV or oral POS and 3 had their crushed POS DRT dose modified.

**Figure:**
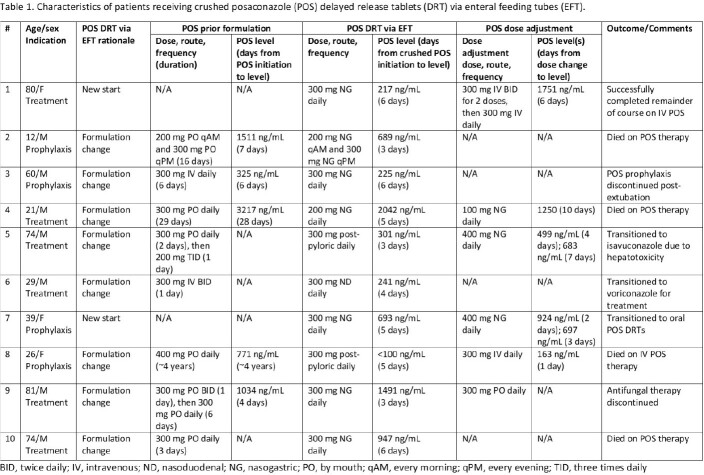

**Figure:**
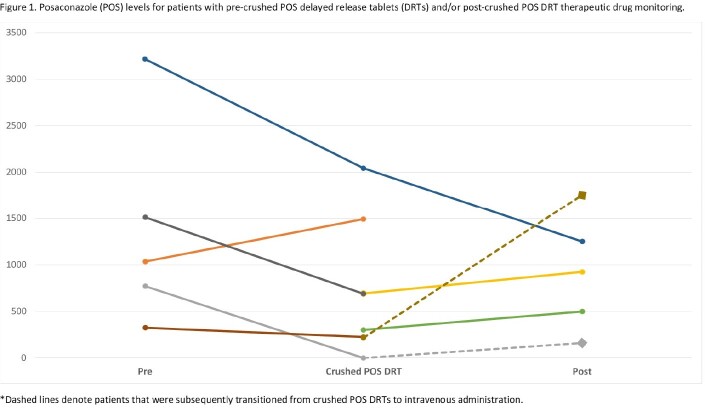

**Conclusion:**

Crushed POS DRTs administered via EFTs failed to achieve therapeutic POS levels in 70% of patients. Caution should be used when implementing crushed POS DRTs and internal data should be reviewed if already implemented. Study limitations include small sample size, single-center design, and limited attempts to titrate crushed POS DRT regimens. Future studies should assess the clinical utility of crushed POS DRTs and explore the impact of standardized nursing administration, drug-drug interactions, and enteral feeding interactions/routes on crushed POS DRT pharmacokinetics.

**Disclosures:**

**All Authors**: No reported disclosures

